# Egg Production in Poultry Farming Is Improved by Probiotic Bacteria

**DOI:** 10.3389/fmicb.2019.01042

**Published:** 2019-05-24

**Authors:** Juan Manuel Peralta-Sánchez, Antonio Manuel Martín-Platero, Juan José Ariza-Romero, Miguel Rabelo-Ruiz, María Jesús Zurita-González, Alberto Baños, Sonia María Rodríguez-Ruano, Mercedes Maqueda, Eva Valdivia, Manuel Martínez-Bueno

**Affiliations:** ^1^Estación Biológica de Doñana – Consejo Superior de Investigaciones Científicas, Seville, Spain; ^2^Departamento de Microbiología, Universidad de Granada, Granada, Spain; ^3^Departamento de Microbiología y Biotecnología – DMC Research Center, Granada, Spain; ^4^Faculty of Science, University of South Bohemia, České Budějovice, Czechia; ^5^Instituto de Biotecnología, Universidad de Granada, Granada, Spain

**Keywords:** bacterial community, egg production, *Enterococcus faecalis* UGRA10, high-throughput sequencing, laying hens, probiotics

## Abstract

Antimicrobial resistance (AMR) is one of the most serious threats for human health in the near future. Livestock has played an important role in the appearance of antibiotic-resistant bacteria, intestinal dysbiosis in farming animals, or the spread of AMR among pathogenic bacteria of human concern. The development of alternatives like probiotics is focused on maintaining or improving production levels while diminishing these negative effects of antibiotics. To this end, we supplied the potential probiotic *Enterococcus faecalis* UGRA10 in the diet of laying hens at a final concentration of 10^8^ Colony Forming Units per gram (CFU/g) of fodder. Its effects have been analyzed by: (i) investigating the response of the ileum and caecum microbiome; and (ii) analyzing the outcome on eggs production. During the second half of the experimental period (40 to 76 days), hens fed *E. faecalis* UGRA10 maintained egg production, while control animals dropped egg production. Supplementation diet with *E. faecalis* UGRA10 significantly increased ileum and caecum bacterial diversity (higher bacterial operational taxonomic unit richness and Faith’s diversity index) of laying hens, with animals fed the same diet showing a higher similarity in microbial composition. These results point out to the beneficial effects of *E. faecalis* UGRA10 in egg production. Future experiments are necessary to unveil the underlying mechanisms that mediate the positive response of animals to this treatment.

## Introduction

The emergence of antimicrobial resistance (AMR) is a worldwide issue in public health (Ferri et al., 2017; World Health Organization, 2018). This situation has been reached due to the abusive prescription of antibiotics, their inappropriate use by patients, and the abuse of these substances in livestock (Hecker et al., 2003; Levy and Marshall, 2004; Capita and Alonso-Calleja, 2013). The indiscriminate use of antibiotics in livestock is due to two major reasons. The first and most obvious one is disease control associated with intensive farming and animal overcrowding (McEwen and Fedorka-Cray, 2002; Gilchrist et al., 2007; Marshall and Levy, 2011). The second reason is the discovery of the effects of antibiotics as growth promoters (AGPs, reviewed in McEwen and Fedorka-Cray, 2002; Dibner and Richards, 2005). Although the mechanisms of action are still unclear, antibiotic imbalance alters the bacterial communities of the intestine, causes alterations in the digestive tract and in the metabolic processes, and increases nutrient absorption (Dibner and Richards, 2005; Miles et al., 2006; Niewold, 2007). In addition, antibiotics affect the immune system, via changes in bacterial community or directly altering the immune response (Bode et al., 2014; Yang et al., 2017). Therefore, one of the bacterial communities most affected by the use of antibiotics is the intestinal microbiome (McEwen and Fedorka-Cray, 2002; Wegener, 2003). These bacteria play a fundamental role in gut homeostasis, balance, and resilience (Clemente et al., 2012; Huttenhower et al., 2012). However, the appearance of undesirable collateral effects, especially affecting the distribution and selection of AMR genes in commensal bacteria, makes these bacteria risky to human health (Wegener, 2003) and makes it necessary to search alternatives to the use of antibiotics in livestock (Ferket, 2004).

Antibiotics have played a major role in maximizing poultry production (McEwen and Fedorka-Cray, 2002; Dibner and Richards, 2005). Thus, they have been used as growth promoters in broilers (Gadde et al., 2018; Wealleans et al., 2018), or egg production enhancers in laying hens (Park et al., 2016). This increase in productivity has been associated with the beneficial role of antibiotics in infection control in poultry farms (Gustafson and Bowen, 1997; McEwen and Fedorka-Cray, 2002; Singer and Hofacre, 2006). However, undesirable and collateral effects have appeared, especially affecting changes in the distribution and selection of AMR genes in commensal bacteria (Teuber, 2001; Diarra et al., 2007; Diarrassouba et al., 2007; Gyles, 2008). Some evidences point out the appearance of AMR in relation to the use of antibiotics in poultry. The relationship between the use of fluoroquinolones and AMR in *Campylobacter* sp. has been evidenced (Alfredson and Korolik, 2007; Nelson et al., 2007). Multi-resistant strains of *Escherichia coli* have been associated with avian farms and have been found in chicken-derived products (Diarra et al., 2007; Diarrassouba et al., 2007; Thibodeau et al., 2007; Gyles, 2008; Osman et al., 2018). Several studies pointed out poultry and derivate-food as a reservoir and potential resource for *Salmonella* sp. resistant strains (Carramiñana et al., 2004; de Oliveira et al., 2005; Singh et al., 2010; Velasquez et al., 2018). Under this scenario of AMR, governments and institutions started to ban the use of antibiotics in livestock (European Commission, 2003, 2005; U.S. FDA, 2017). However, the prohibition of antibiotics in livestock in general, and in the poultry sector in particular, has caused an increase in incidence of infectious diseases by *Campylobacter jejuni* or *Clostridium perfringens* (reviewed in Van Immerseel et al., 2004; Alfredson and Korolik, 2007). Therefore, there is considerable concern and a certain need to replace antibiotics in disease control and as growth promoters (Joerger, 2003; Griggs and Jacob, 2005).

Different agents have been suggested to substitute antibiotics in poultry farming, such as prebiotics and probiotics (Patterson and Burkholder, 2003; Gaggia et al., 2010). Most probiotics used in aviculture are bacteria that already exist in the digestive tract of animals and have properties of interest as signal modulators of intestinal cells, bacterial community stabilizers or competitors against undesirable bacterial species (de Vrese and Schrezenmeir, 2008; Kabir, 2009). Although *Enterococcus* species are not “generally recognized as safe” (GRAS, reviewed in Ogier and Serror, 2008), different *Enterococcus* species have been tested as probiotics (Fuller, 1989; Franz et al., 2011). The use of enterococci as probiotics remains controversial: while the probiotic benefits of some strains have been well-established, the increase in enterococcal diseases associated with human health and resistance to multiple antibiotics has raised concerns about their use (Franz et al., 2003). Despite this controversy, some strains are commercialized as probiotics, such as *Enterococcus faecium* SF68 (Cylactin, F. Hoffmann-Roche, S.A., Switzerland) and *Enterococcus faecalis* (Symbioflor, SymbioPharm, Germany) (Habermann et al., 2001; Fenimore et al., 2017; Torres-Henderson et al., 2017). *E. faecalis* has been pointed out as a potential substitute for antibiotics (Gaggia et al., 2010). *E. faecalis* is a common bacterium in vertebrate gut, Gram+, facultative anaerobe, with a high degree of tolerance to pH (3–10) and salinity [6.5% NaCl (w/v)] (Krieg and Holt, 1984; Schleifer and Kilpper-Bälz, 1984) which would allow gastrointestinal transit to the large intestine. Moreover, they are good producers of antagonistic substances, especially bacteriocins, also called enterocins, which allow a great interaction with the rest of the community (Foulquie-Moreno et al., 2006; Martín-Platero et al., 2006; Trmcic et al., 2011). In this sense, the strain *E. faecalis* UGRA10 isolated from an Andalusian goat cheese is a producer of the enterocin AS-48 and has very interesting technological properties: it is resistant to high concentrations of bile [up to 40% (w/v)]; shows a high antagonistic spectrum including Gram- and Gram+ bacteria (Cebrian et al., 2012) and seems to stimulate immune response in animals (Baños, 2016). Interestingly, this strain is harmless to mice and fish, and protects against some pathogenic strains such as *C. perfringens* in mice and *Lactococcus garvieae* in rainbow trout and zebrafish (Baños, 2016). These properties make *E. faecalis* UGRA10 an excellent candidate for probiotic and a model to test its implantation, its effects on the community and, especially, on health and production of animals.

We investigated the possible influence of *E. faecalis* UGRA10 on egg production and gut microbiome of laying hens when administered in the diet, combining the classical culture techniques with the latest high throughput sequencing.

## Materials and Methods

### Laying Hens and Farm Facilities

The experiment was performed at Granja Avícola Gil, SL, a laying hen farm (Alhendín, Granada, Spain). Laying hens were kept at 20 ± 2°C and 78 ± 3% relative humidity (average ± standard deviation), under a photoperiod of 16 h per day. The farm fulfilled the national regulations and the European directive for the protection of animal welfare in research (Directive 2010/63/EU, European Commission, 2010).

### Experimental Design and Sampling Collection

Three production lines housed 180 experimental laying hens in groups of 6 hens per cage, with food and water *ad libitum*. All laying hens belonged to the same Hy Line brown variety and were placed in cages at the age of 16 weeks. Cage distribution between treatment groups was randomly assigned in three production lines. Hens were kept and fed during 2 weeks for acclimation.

Control hens (90 hens, 15 cages) received a basal fodder diet (45% fish flour, 35% soya, 8% granulated corn, 7% bran corn, and 5% sunflower bread) while experimental hens (90 hens, 15 cages) received the same diet but supplemented with the bacterium *E. faecalis* UGRA10 (see below).

Hens were first kept at the farm for 15 days for acclimation. Experiment started on April 8^th^, and egg production (number of eggs) of each treatment group was recorded every working day until the day 76. At this experimental farm, laying hens are slaughtered after 76 days. One day per week, eggs from each treatment were weighted and classified into size categories (S: <53 g; M: 53–63 g; L: 63–73 g; XL; >73 g) according to EU regulation (European Commission, 2008). Fecal samples were collected on days 7, 15, 40, and 76 of the experiment. On day 40, 13 hens of each group, they were euthanized by an intrathoracic injection of 2 mL/hen of the euthanasic T-61 (Intervet, Salamanca, Spain). On day 76, 10 hens from each group were euthanized following similar procedure. Immediately after slaughtering, hens were dissected and ileum and caecum were collected with sterile material, kept in sterile plastic bags, and transported directly to the lab where samples were processed. No animal died during the experimental period due to illness or malnutrition.

### *E. faecalis* UGRA10 Production

*Enterococcus faecalis* UGRA10 was isolated by the Microbial Antagonism research group from the University of Granada (Cebrian et al., 2012). The bacteria were cultured in bioreactors in the DMC Research facilities located in Alhendín (Granada, Spain), as by-product of AS-48 bacteriocin production. Briefly, the strain was cultured from -80°C stock in Tryptic Soy Agar (Scharlau, S.L., Spain) and isolated colonies were inoculated into 2 L Brain Heart Infusion broth (BHI, Scharlau, S.L., Spain) and incubated at 28°C for 24 h. This primary inoculum was added (5%) to a broth based on Lactoalbumin Esprion 300 (E-300, DMW International, Holland) as described in Ananou et al. (2008) in a 20 L biofermenter (Biobech 20 Applikon Biotechnology, Delft, Netherlands), and then incubated at 28°C for 24 h. Afterward, 16 L of this culture were added of a 300 L of Lactoalbumin Esprion 300 in a CHEMAP fermenter (Compact Unit, Process Engineering Company, India) and incubated at 29°C for 24 h. All inocula and broths were adjusted at pH 6.5. Cells were collected by means of aM-500 ultra-filtration equipment (BionetIngenieria, Spain). Afterward, cells were suspended in saline buffer and re-centrifuged twice. Final clean pellets were kept at -20°C.

Cells were encapsulated into β-cyclodextrin microstructures and kept at 4°C for up to 15 days. β-cyclodextrin is an excellent transport agent due to the lack of significant effects on hosts (Del Valle, 2004). In order to study cell viability included inside β-cyclodextrin, we cultured serial decimal dilutions of three samples of these microstructures (1 g of sample diluted in 10 mL of phosphate buffer) at different time intervals (0, 7, 15, and 30 days of storage) in TSA and incubated at 37°C for 48 h. Cells viability was reduced to 15–30 days of storage (Kruskal–Wallis, H_2,11_ = 9.36, *P* = 0.025). Following a conservative strategy, β-cyclodextrin complex was produced every 15 days in order to ensure cell viability. UGRA10-β-cyclodextrin microstructures were mixed with the food of experimental laying hens at a final concentration of 10^8^ Colony Forming Units per gram (CFU/g) of fodder. Probiotic was administered daily throughout the experiment.

### Enumeration of Microorganisms in Fecal Samples

Once in the lab, fecal samples were weighted, transferred to lab blender bags, and diluted 10-fold in phosphate saline buffer added with 0.5 g/L cysteine hydrochloride (for ensuring viability of anaerobic bacteria). The samples were homogenized using a stomacher lab blender (IUL Instruments, Spain) for 2 min. For each fecal treatment and sampling point (7, 15, 40, and 76 days), three sets of samples were collected. Each set consisted of a mixture of seven fecal samples from different cages with the same treatment.

Decimal serial dilutions of each set were performed and cultured in triplicate on Wilkins-Chalgren agar for total anaerobic bacteria (Scharlau, S.L., Spain) and Slanetz-Bartley agar for *Enterococcus* sp. (Scharlau, S.L., Spain). Plates were incubated at 37°C for 48 h in 2.5 L anaerobic chambers (Oxoid) and 2.5 L AnaeroGen Compact system (Thermo Scientific). Bacterial counts were expressed as CFU/g per gram of fecal sample.

### *E. faecalis* UGRA10 Indirect Detection in Fecal Samples

We tested the inhibition capacity of isolates from fecal samples against two indicator strains, *E. faecalis* S-47 and *E. faecalis* UGRA10. We expected the *Enterococcus* population in feces of the treated group to be dominated by *E. faecalis* UGRA10, so most of the isolates will produce inhibition halos against S-47 (Cebrian et al., 2012). However, these halos will disappear in the presence of the original strain due to the fact that *E. faecalis* UGRA10 is immune to its own bacteriocin (Cebrian et al., 2012).

Indicator strains were cultured from the stock of the Lactic Acid Bacteria Laboratory in the University of Granada. After isolation in Brain Heart Infusion agar (BHA), colonies were cultured overnight in 6 mL BHI. The antagonistic activity was tested following Tagg and Mcgiven (1971). Stainless steel cylinders for antibiotics (diameter: 8 mm, height: 10 mm; Scharlab, S.L.) were placed on a layer of 10 mL Müller-Hilton agar (Scharlau, S.L.) buffered with phosphate buffer saline (pH 7.2, *M* = 0.2). Afterward, a 6 mL of BHA (Scharlau, S.L.) tempered around 55°C, were inoculated with one of the indicator strains (around 10^8^ CFU per mL), shaken in a vortex and extended over the Müller-Hilton agar. Once the BHA solidified, the cylinders were taken out, leaving a circular hole in the BHA layer.

From Slanetz-Bartley agar plates, we selected 320 colonies randomly from plates for each treatment and sampling (2 treatments × 4 sampling times × 40 colonies). Each colony was incubated overnight in tubes containing 6 mL of BHI tubes, 1 mL was centrifuged and 100 μL of supernatant were added to the holes. Plates were incubated during 18–24 h at 37°C. Inhibition activity was measured as presence or absence of inhibition halo.

### High-Throughput Sequencing

Bacterial total DNA from ileum and caecum samples was extracted by following the Modified Salting-Out Procedure by Martín-Platero et al. (2007). Amplicon PCR was performed from bacterial total DNA on the V4 region of the 16S rRNA gene by using the primer pair 515f (5′-GTGCCAGCMGCCGCGGTAA-3′) – 786r (5′-GGACTACHVGGGTWTCTAAT-3′) with Golay barcodes on the forward primer. High-throughput sequencing was performed on Illumina Miseq platform in the Scientific Instrumental Center at the University of Granada (Spain). Ileum and caecum of 5 control and 10 treated hens were used in subsequent analyses, for both sampling times (on days 40 and 76). Six samples failed to amplify (see distribution in Supplementary Table S1). Sequences are available in the Sequence Read Archive (SRA) in the GenBank – NCBI webpage^1^ under Accession Nos. SAMN09603288 to SAMN9603361.

Subsequent analyses were performed with QIIME2 v2018.02 (Quantitive Insights In Microbial Ecology, Caporaso et al., 2010). Primer trimming, pair joining, and quality filtering were performed by using default parameters. Afterward, we used Deblur, a sub-operational-taxonomic-unit (sOTU) approach, in order to remove sequencing errors (Amir et al., 2017). We used the fragment insertion script implemented in QIIME2, a script that performs the sequence alignment and *de novo* phylogenetic tree (Janssen et al., 2018). Taxonomy assignment was based on Greengenes 13_08 with a similarity of 99% (DeSantis et al., 2006). Finally, chloroplasts and mitochondria were removed from the sOTU table, but Cyanobacteria were retained in subsequent analyses (Ley et al., 2005).

### Statistical Analysis

We used general linear models (GLMs) to explore the effect of the treatment, sampling date and their interaction in different dependent variables: bacterial cultures, number of eggs (Supplementary Table S2), and different indexes of alpha diversity. We also explored the effects of egg size as factor (S, M, L, or XL as describe above). Whitaker (1972) defined diversity in three different levels: alpha diversity is the diversity found in a sample; beta diversity is the compositional difference between samples; and gamma diversity is the diversity at the regional scale. We calculated three different alpha diversity indexes from the sOTU table: bacterial operational taxonomic unit (OTU) richness (or number of observed OTUs), Faith’s phylogenetic diversity index (Faith and Baker, 2006) and chao1 (Chao, 1984). Residuals of the dependent variables after analyses followed normal distribution (Kolmogorov–Smirnov normality test; *P* > 0.20) and were homoscedastic (Levene’s test for homogeneity of variances, all *P* > 0.19). These results validate the use of parametric statistical tests. These analyses were performed in Statistica 10.0.

Beta diversity distance matrixes were calculated using Unifrac distance (Lozupone and Knight, 2005) based on a rarefied sOTU table at 1800 sequences depth per sample. Both weighted and unweighted Unifrac distance matrixes were used in subsequent analyses as we do not have *a priori* predictions in the effects of the independent variables (treatment, sampling date, and gut portion) on the bacterial community. Weighted Unifrac gives more importance to the most abundant bacteria as it takes into account sequence abundance per sOTU, while unweighted Unifrac gives similar weight to all bacterial sOTU present in the samples, i.e., it gives more importance to the minority bacteria as it takes into account the presence or absence of a sOTU. Procrustes ANOVA was used to test these effects on both Unifrac distance matrixes (Collyer et al., 2015), using the geomorph (Adams and Otarola-Castillo, 2013) and vegan (Oksanen et al., 2016) packages. Principal Coordinate Analyses were performed and visualizations of the three first PCoA axes were plotted using Emperor 2018.2.0 (Vazquez-Baeza et al., 2013).

## Results

### Alpha Diversity of Bacterial Community in Ileum and Caecum

**FIGURE 1:**
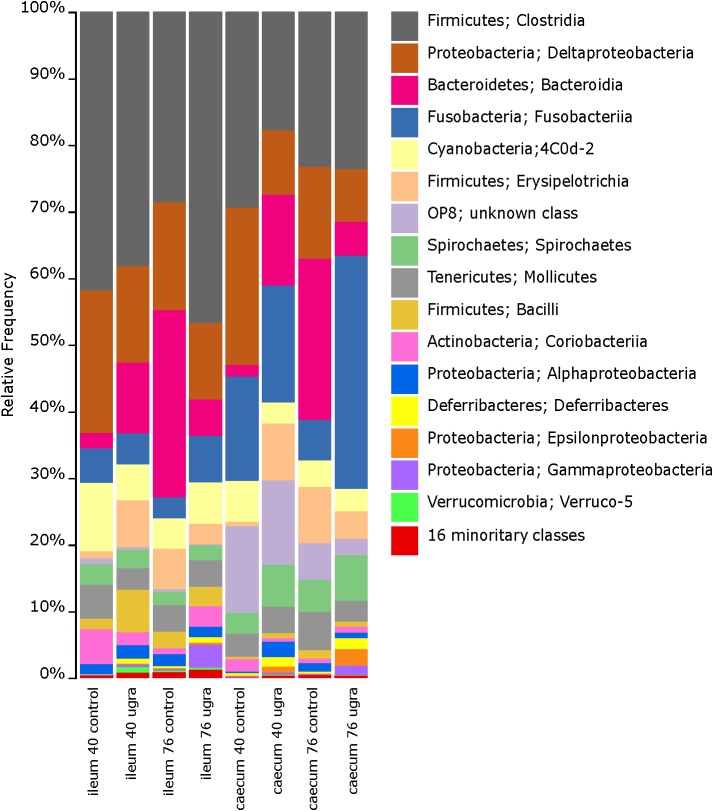
Bar plots of average relative bacterial abundance in gut regions of laying hens at the class level, grouped by sampling time and treatment. Classes in the legend are sorted by sequence relative abundance, from most abundant to least abundant. UGRA refers to hen fed *Enterococcus faecalis* UGRA10 at 10^8^ CFU per gram of fodder per day; while 40 and 76 refers to the number of days after the experiment started.

Ileum microbiome of control hens on both 40 and 76 days were dominated at the class level by *Clostridia, Bacteroidia, Erysipelotrichi, Mollicutes, Deltaproteobacteria*, and *Bacilli* (Figure 1 and Supplementary Figure S1). These dominant classes were present in *E. faecalis* UGRA10 treated hens on day 40, although proportions of each class changed, being the class *Clostridia* the only dominant one (Figure 1 and Supplementary Figure S1). The ileum community in the control hens was very diverse at the genus level, dominated by *Desulfovibrio, Bacteroides*, an unknown genus of the order *Bacteroidales*, an unknown genus of the family *Ruminococcaceae* and an unknown genus of the family *Mogibacteriaceae* (Supplementary Figure S2). The bacterial community of samples from hens treated with *E. faecalis* UGRA10 was very similar, although other dominant genus as *Phascolarctobacterium* or *Megamonas* appeared (Supplementary Figure S2).

**FIGURE 2:**
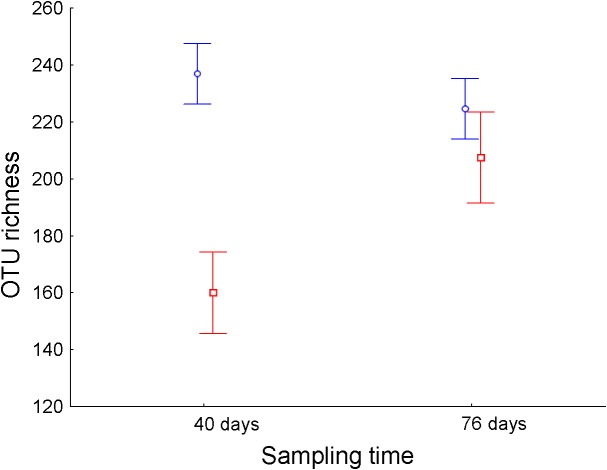
Average ± standard error of the mean of the bacterial OTU richness (number of different OTUs) of ileum of laying hens at different sampling times (in days) from control (*n* = 5, red) and hens supplemented with *E. faeca*lis UGRA10 (*n* = 10, blue).

Operational taxonomic unit richness in the ileum differed significantly between treatments throughout the experimental period (Table 1). While *E. faecalis* UGRA10 group kept similar levels of bacterial OTU richness, the control one experienced an increase in OTU richness until reaching similar levels to those of the treatment group at day 76 (Figure 2 and Supplementary Table S3). Similar pattern was marginally significant in the case of Faith’s diversity index (see interaction term in Faith’s diversity index in ileum, Table 1).

**Table 1:** General linear Models exploring the effects of treatment (control and *E. faecalis* UGRA10 administration) and sampling time (days 40 and 76) in the different alpha diversity indexes of the bacterial community of ileum and caecum of laying hens.

	Explanatory variables	d.f.	*F*	*P*
**Ileum**				
Species richness	Treatment	1,23	12.83	**0.002**
	Time	1,23	1.79	0.194
	Treatment × Time	1,23	5.18	**0.032**
Pielou evenness	Treatment	1,23	0.27	0.608
	Time	1,23	5.32	**0.030**
	Treatment × Time	1,23	1.52	0.230
Faith’s diversity index	Treatment	1,23	10.70	**0.003**
	Time	1,23	1.40	0.249
	Treatment × Time	1,23	4.08	0.055
Shannon’s diversity index	Treatment	1,23	1.24	0.277
	Time	1,23	1.60	0.219
	Treatment × Time	1,23	0.01	0.987
**Caecum**				
Species richness	Treatment	1,23	0.23	0.636
	Time	1,23	0.41	0.528
	Treatment × Time	1,23	0.00	0.966
Pielou evenness	Treatment	1,23	0.23	0.638
	Time	1,23	0.84	0.370
	Treatment × Time	1,23	0.41	0.529
Faith’s diversity index	Treatment	1,23	0.72	0.405
	Time	1,23	1.21	0.283
	Treatment × Time	1,23	0.35	0.561
Shannon’s diversity index	Treatment	1,23	0.26	0.618
	Time	1,23	0.29	0.597
	Treatment × Time	1,23	0.21	0.650


*D.f. refers to degree of freedom. Significant *P*-values are shown in bold.*

The bacterial community of caecum was more diverse than that of ileum (Figure 1 and Supplementary Figure S1). Major classes on days 40 and 76 in both experimental groups included *Clostridia, Deltaproteobacteria, Bacteroidia* and the unknown phylum OP8 (Figure 1 and Supplementary Figure S1). The genera abundance between treatments and sampling date followed similar patterns. Bacterial community was dominated by *Phascolarctobacterium, Fusobacterium, Desulfovibrio*, an unknown genera belonging to the class Cyanobacteria and *Megamonas* (Supplementary Figures S2A,B). Alpha diversity was very similar between treatment groups, showing no significant changes throughout the experimental period (Table 1).

### Effects of Treatment and Sampling Date on Beta Diversity

**FIGURE 3:**
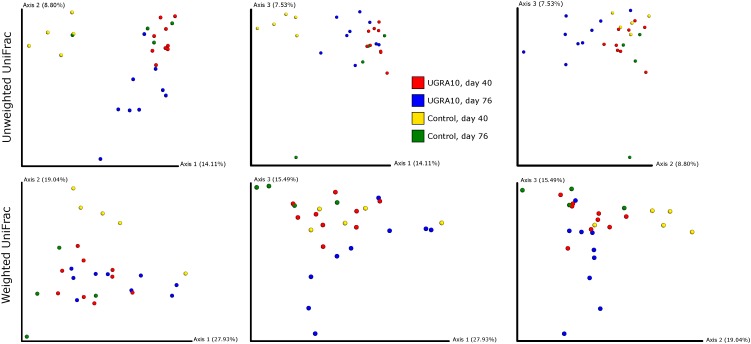
Two-dimensional figures showing three first axes of Principal Coordinate Analysis and representing bacterial communities of ileum of laying hens, using Unweighted and Weighted Unifrac distance matrixes. Proportion of explained variance by each PCo axes is also shown.

In the ileum region, changes of bacterial communities throughout the experiment period varied from one treatment to another (see Figure 3 and the interaction term in Table 2). Interestingly, control communities on day 76 overlapped with bacterial communities of *E. faecalis* UGRA10 treatment.

**Table 2:** PROCRUTES ANOVA exploring the effects of treatment, sampling date and their interaction in the bacterial community of laying hens fed with a control diet or supplemented with *Enterococcus faecalis* UGRA10.

	β-Diversity distance matrix	Explanatory variables	d.f.	*F*	*P*
Ileum	Unweighted Unifrac	Day	1,23	1.47	**0.020**
		Treatment	1,23	2.48	**0.001**
		Day × Treatment	1,23	2.57	**0.001**
	Weighted Unifrac	Day	1,23	1.63	0.060
		Treatment	1,23	2.04	**0.026**
		Day × Treatment	1,23	4.16	**0.001**
Caecum	Unweighted Unifrac	Day	1,23	1.44	**0.029**
		Treatment	1,23	2.12	**0.004**
		Day × Treatment	1,23	2.06	**0.003**
	Weighted Unifrac	Day	1,23	1.54	0.105
		Treatment	1,23	2.32	**0.024**
		Day × Treatment	1,23	2.60	**0.006**


*D.f. refers to degree of freedom. Significant *P*-values are shown in bold.*

**FIGURE 4:**
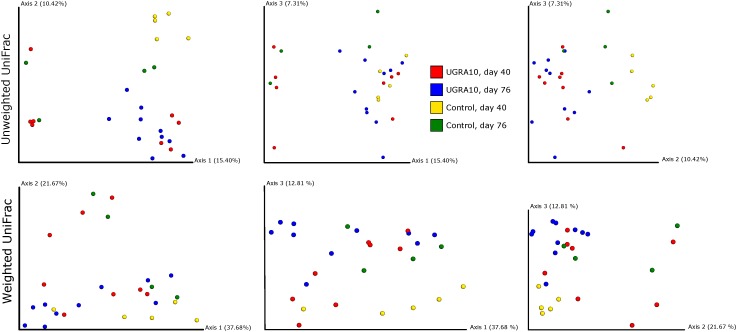
Two-dimensional figures showing three first axes of Principal Coordinate Analysis representing bacterial communities of caecum of laying hens, using Unweighted and Weighted Unifrac distance matrixes. Proportion of explained variance by each PCo axes is also shown.

Treatment explained a significant proportion of the variance in bacterial community of caecum (both weighted and unweighted Unifrac) and its effect depends on the sampling date (see interaction term in Figure 4 and Table 2).

### Presence of *E. faecalis* in Feces

Cultures from each sample were highly and significantly correlated within each triplicate (Kruskal–Wallis, bacterial count as dependent variable, sample as factor, H_23,72_ = 62.44, *P* < 0.001), so we calculated the average values for each set of samples within each sample date and each bacterial culture.

*Enterococcus* sp. was successfully recovered from all Slanetz-Bartley agar plates [control, *N* = 72, log10 CFUs = 6.55 ± 0.16 (average ± SE); *E. faecalis* UGRA10: *N* = 72, 6.67 ± 0.20]. However, treatment, sampling date or their interactions did not significantly explain variation in bacterial counts of *Enterococcus* sp. (GLM, average bacterial count, treatment as factor, *F*_1,20_ = 0.01, *P* = 0.915, sampling time as covariable, *F*_1,20_ = 2.15, *P* = 0.158, interaction, *F*_1,20_ = 0.13, *P* = 0.721).

Following a similar pattern, total anaerobic bacteria (control, *N* = 72, log10 CFUs = 7.27 ± 0.99; *E. faecalis* UGRA10: *N* = 72, 7.40 ± 0.86) did not differ between treatments, sampling date or their interaction (GLM, average bacterial count, treatment as factor, *F*_1,20_ = 0.05, *P* = 0.827, sampling time as covariable, *F*_1,20_ = 0.58, *P* = 0.454, interaction, *F*_1,20_< 0.01, *P* = 0.990).

### *E. faecalis* UGRA10 Indirect Detection in Feces

The percentage of colonies that showed inhibition properties against *E. faecalis* S-47 increased along the experiment but only in the treatment group. In the control group, a low percentage of colonies showed antagonism against S-47 (Table 3). Interestingly, most of the colonies that produced antagonism against *E. faecalis* S-47 in the treatment group did not show inhibition against *E. faecalis* UGRA10, except 1 out of 30 colonies on day 80. Most of these colonies may be attributed to *E. faecalis* UGRA10, as this strain is immune against its own bacteriocin (Table 3).

**Table 3:** Percentages of colonies from fecal samples of laying hens that showed inhibition against indicators strain *E. faecali*s S-47 and percentage of those colonies that inhibition halo disappear against *E. faecalis* UGRA10.

Sampling time	Treatment	% inhibitor colonies against S-47	% of colonies where inhibition halo disappeared against UGRA10
7	Control	0.0	0.0
	UGRA10	0.0	0.0
15	Control	2.5	0. 0
	UGRA10	10.0	100.0
38	Control	0.0	0.0
	UGRA10	37.5	100.0
80	Control	2.5	0.0
	UGRA10	75.0	96.7


### Egg Production

**FIGURE 5:**
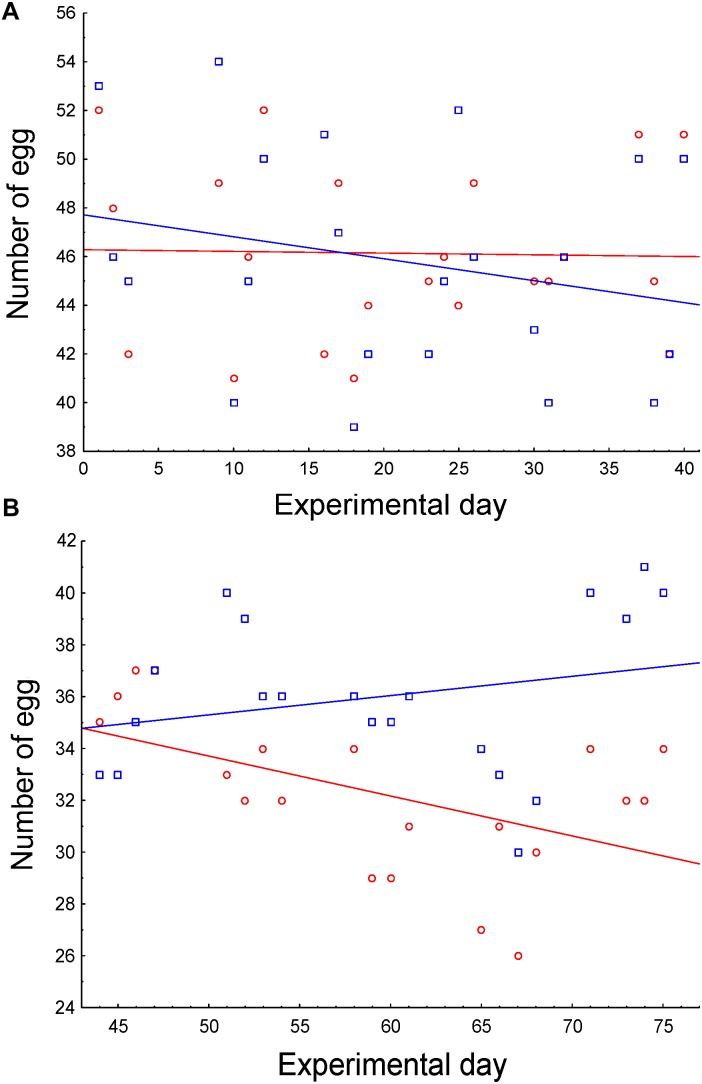
Correlations between number of eggs and sampling date as function of treatment, during the first **(A)** and second **(B)** half of the experimental period. Control hens (red) were fed a basal diet while experimental hens (blue) were supplemented with *E. faecalis* UGRA10.

As 13 hens were slaughtered on day 40, we analyzed both periods separately, before and after slaughtering. Egg production was maintained in the first half of the experiment, regardless of the treatment (Figure 5A and Table 4), as both slopes of egg production did not differ from 0 (control slope = -0.007, *r* = -0.02, *P* > 0.915; experimental slope = -0.09; *r* = -0.24; *P* = 0.277). However, egg production along the second period significantly differed between treatments. While egg production of control hens significantly decreased (slope = -0.15, *r* = -0.51, *P* = 0.020), hens supplemented with *E. faecalis* UGRA10 maintained egg production along this period (slope = 0.07, *r* = 0.24, *P* = 0.293; Figure 5B and Table 4).

**Table 4:** General Linear Models explaining egg production per laying hen in both control and diet supplemented with *Enterococcus faecalis* UGRA10, during both halves of the experimental period.

	Explanatory variables	d.f.	*F*	*P*
First half	Treatment	1,40	0.65	0.425
	Sampling date	1,40	0.88	0.353
	Treatment × Sampling date	1,40	0.65	0.425
Second half	Treatment	1,36	6.22	**0.017**
	Sampling date	1,36	0.76	0.390
	Treatment × Sampling date	1,36	6.22	**0.017**


*D.f. refers to degree of freedom. Significant results are in bold.*

**FIGURE 6:**
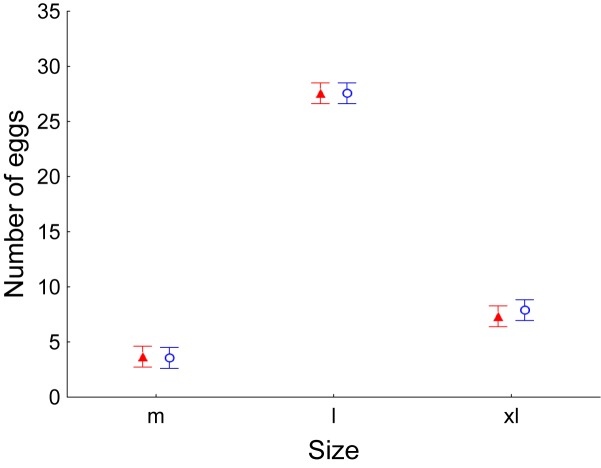
Average ± Standard error of the mean number of eggs produced by laying hens. Once per week, number of eggs was recorded as well as their weight and their size (m: medium; l: large; xl: extra-large). Control values are shown in red while values of laying hens supplemented with *E. faecalis* UGRA10 in the diet are shown in blue.

Laying hens throughout the experiment period produced significantly more eggs of size L (between 63 and 73 g), regardless of the treatment (GLM, number of eggs as dependent variable, Treatment as factor, *F*_1,47_ = 0.04, *P* = 0.848, Egg size as factor, *F*_2,47_ = 370.69, *P* < 0.001, Sampling date as covariable, *F*_1,47_ = 15.03, *P* < 0.001, Interaction between egg size and treatment, *F*_2,47_ = 0.07, *P* = 0.931; Figure 6). Interestingly, no hen produced any egg of size S (less than 53 g).

## Discussion

The addition of *E. fae*calis UGRA10 in the diet of laying hens had a positive and significant effect on egg production during the second half of the experiment, allowing these hens to maintain production levels throughout their productive lives. These beneficial productive changes were accompanied by significant changes in the gut microbiota and an increase in the presence of *E. faecalis* UGRA10 along the experimental period in the feces. These results support the use of *E. faecalis* UGRA10 as an enhancer of egg production in laying hens, while modifying bacterial community diversity.

Maintenance of egg production levels throughout laying hens’ welfare is essential in poultry and the use of probiotics is one of the most promising strategies to achieve this goal (Patterson and Burkholder, 2003; Kabir, 2009). Our results support this strategy as treated hens maintained egg production, especially during the second half of the experimental period, without altering egg size. In fact, large size eggs (L: 63–73 g; and XL: >73 g) were the most produced eggs. These results are really promising as large size eggs are most demanded in different markets (Araneda Uson, 2006; Souza, 2008; Bejaei, 2009; Reichmann Bellino and Arias Nieto, 2016; Zakowska-Biemans and Tekien, 2017). Different bacterial strains have been studied and their effects tested on animal performance, egg production, enhancement of immune system and changes in gut microbiome. For instance, *Rhodobacter capsulatus* improved hen health and egg quality during the last period of the laying (Lokhande et al., 2013); *Bacillus subtilis* has been tested successfully against *Salmonella* infection (Oh et al., 2017) and increased egg quality and production (Ribeiro et al., 2014); or *B. licheniformis* acted as an immune system enhancer and a hormone regulator (Wang et al., 2017). The use of *E. faecalis* UGRA10 showed some advantages as enterococci are common bacteria in warm-blood animals (Krieg and Holt, 1984) and have a beneficial interaction with immune system (Franz et al., 2011), hormones and metabolism (Zhao et al., 2013) and gut microbiota (Han et al., 2013; Park et al., 2016). We recovered a high proportion of *Enterococcus* sp. in all fecal samples, around one logarithmic unit smaller than total anaerobic bacteria, so this taxon is well-represented in those fecal samples. Interestingly, the presence of *E. faecalis* UGRA10 increased during the experimental period in the treated group, until it became dominant in fecal samples. This result suggests that this strain may substitute other *Enterococcus* strains/species. This substitution between *Enterococcus* species has been reported before (Sakai et al., 2006; Saelim et al., 2012) and may be caused by the production of antagonistic substances against closely related species (Foulquie Moreno et al., 2003, 2006; Martín-Platero et al., 2006, 2009; Ruiz-Rodríguez et al., 2013). In this sense, the ability of allochthonous bacteria, such as probiotics, to establish and flourish in complex bacterial ecosystems (as the intestine of vertebrates) is low and depends on taxon (Thomas and Versalovic, 2010). However, the positive effects of these transient bacteria are related to the active and temporary interaction with the host immune system (Are et al., 2008; Thomas and Versalovic, 2010), even though they do not establish as resident members of the bacterial community (Derrien and Vlieg, 2015). For instance, *Enterococcus* as probiotic in poultry seems to induce physical changes in the gut structure, especially in the development of villus height/crypt depth ratio and villus height in the ileum (Awad et al., 2008), hence the increase in nutrient digestibility (Park et al., 2016). An alternative and non-exclusive hypothesis would point out the role of enterococci as immune system stimulators (Franz et al., 2011) or hormone mediators in avian performance (Zhao et al., 2013). These interactions with the immune system have been examined in model organisms (Foligne et al., 2007; de Vrese and Schrezenmeir, 2008), although our knowledge of this interaction in poultry is still scarce. In broilers, probiotic supplementation in the diet increases serum/plasma immunoglobulin levelsandfoster change in immune cell numbers and their phagocytosis capacities (Apata, 2008; Lee et al., 2011; Zhang et al., 2012; Salim et al., 2013; Ahmed et al., 2014; Beirao et al., 2018). The use of *E. faecium* as a probiotic produces increases in antibody titers against pathogens are also found (Nayebpor et al., 2007), changes inliver metabolic efficiency (Zheng et al., 2016), modifications in intestinal mucosa proteome (Luo et al., 2013) or increases in leptin levels and hence growth rate (Arslan et al., 2004). In laying hens, a combination of probiotics enhanced antibody response (Zhang et al., 2012). However, the mechanisms that explain the interaction between the use of *Enterococcus* as probiotic and the immune system and/or hormones in laying hens are still elusive, so further experiments are necessary to elucidate these relationships.

Microbiome of ileum in broilers is dominated by *Lactobacillus*, followed by *Clostridium, Streptococcus*, and *Enterococcus* species (Lu et al., 2003). However, *Clostridium* sp. and closely related species became dominant in caecum of laying hens, followed by members of phylum *Bacteroidetes*, especially *Bacteroides, Butyricimonas*, and *Prevotella* (Callaway et al., 2009; Nordentoft et al., 2011). Our results are consistent with these previous findings, especially at the phylum level, although differ in the importance of different genera. These differences in bacterial community were found mainly in ileum between treatment and sampling time. Treated hens harbored more bacterial species and a wider range of phylogenetic diversity in the ileum than control ones. Nevertheless, bacterial community in caecum showed similar levels of alpha diversity between treatments. These results of alpha diversity are supported with results of beta diversity. Bacterial communities grouped closely when we compared similarities in Unifrac distance matrixes, showing microbiome change in the gut of treated hens.

The beneficial effects of antibiotics on broilers and hens are related to changes in the microbial community of the gut (Choi et al., 2018) especially toward short-chain fatty acid producers (Banerjee et al., 2018), but also to the increase of amino acid metabolites, fatty acids, nucleosides, and vitamins (Gadde et al., 2018). Alternatively, antibiotics improve performance through an anti-inflammatory effect mediated by the intestinal epithelium (Niewold, 2007). In spite of the differences in nature of antibiotics and probiotics, the effects of both agents on animals seem to be similar. *Enterococcus* used as a probiotic in poultry induces shifts of fecal microbiota (Han et al., 2013), especially reducing *Salmonella* populations (Waters et al., 2005), although the major phyla abundance (*Bacteroidetes* and *Firmicutes*) remains stable (Zhao et al., 2013). The diet supplemented with *E. faecalis* UGRA10 produced changes in the bacterial community, both in the ileum and the caecum, and supports, at least partially, the hypothesis that the beneficial effect on the maintenance of egg production could be mediated by effects on the gut microbiome. Further experiments should be conceived in order to unveil possible mechanisms in relation to the stimulation of the immune system or the interaction with hormones.

This experiment supports the use of *E. faecalis* UGRA10 in the diet of laying hens for successfully maintaining egg production levels and change microbiome diversity, similar effects to those found with the use of antibiotics (Park et al., 2016). However, the use of a bacterial strain would avoid the use of antibiotics in poultry, since a new member of the bacterial community is introduced instead of high antibiotic doses during porlongated exposition times.

## Ethics Statement

This study was carried out in accordance with the national regulations and the European directive for the protection of animal welfare in research (Directive 2010/63/EU, European Commission, 2010). This study was performed in a regular poultry farm, following Spanish (Law 32/2007, Real Decreto 348/2000, Real Decreto 3/2002, Real Decreto 372/2003) and European regulation for poultry exploitations (Directive 98/58/CE, European Commission, 1998). We also followed the recommendations provided by the National Ministry of Agriculture, Food and Environment (Ministerio de Agricultura, Alimentación, y Medio Ambiente, Guideless of good practices of animal management and welfare in egg producing farms, 2012).

## Author Contributions

JP-S, AM-P, JA-R, AB, and MM-B conceived and planned the experiments. JA-R and AB carried out the farm experiments. JA-R, MR-R, and MZ-G contributed to sample preparation and performed lab analyses. JP-S, AM-P, JA-R, MR-R, MZ-G, SR-R, MM, EV, and MM-B contributed to the interpretation of the results. JP-S and AM-P took the lead in writing the manuscript. All authors provided critical feedback and helped shape the research, analysis, and manuscript.

## Conflict of Interest Statement

The authors declare that the research was conducted in the absence of any commercial or financial relationships that could be construed as a potential conflict of interest.

## Footnotes

1 https://www.ncbi.nlm.nih.gov/sra/

## Supplementary Material

FIGURE S1 Bar plots of bacterial relative abundance at the class level in laying hens at different gut region, sampling times, and treatments. Classes in the legend are sorted by sequence relative abundance, from most abundant to lowest abundant. UGRA refers to hen fed with *Enterococcus faecalis* UGRA10 at 10^8^ Colony Forming Units per grams of fodder; while 40 and 80 refers to the number of days after the experiment started.

FIGURE S2 Bar plots of relative bacterial abundance at the genus level of ileum and caecum in laying hens at different gut region (ileum and caecum), sampling times (30 and 80 days) and treatments (control and UGRA) **(A)** or grouped by gut region, sampling time and treatment **(B)**. Classes in the legend are sorted by sequence relative abundance. UGRA refers to hen fed with *Enterococcus faecalis* UGRA10 at 10^8^ Colony Forming Units per grams of fodder; while 40 and 80 refers to the number of days after the experiment started.

TABLE S1 Sample sizes of bacterial community of ileum and caecum of laying hens, successfully amplified in Illumina MiSeq platform, at different sampling times. In parentheses, sample size before amplification.

TABLE S2 Number of eggs produced by hens fed with control diets and supplemented with Enterococcus faecalis UGRA10 along the experimental period.

TABLE S3 Summary of average (±SE) of alpha diversity indexes based in bacterial community of ileum and caecum in laying hens sequenced by Illumina Miseq. Different indexes have been separated by gut region, sampling date and treatment. In treated group, laying hens were supplemented with *Enterococcus faecalis* UGRA10. Sample size is also shown.
