# Supplementary material for: Egg Production in Poultry Farming Is Improved by Probiotic Bacteria
Source: Front Microbiol. 2019 May 24;10:1042. doi: 10.3389/fmicb.2019.01042 (PMC6543855; doi:10.3389/fmicb.2019.01042)
Supplement: FIGURE S1 — Bar plots of bacterial relative abundance at the class level in laying hens at different gut region, sampling times, and treatments. Classes in the legend are sorted by sequence relative abundance, from most abundant to lowest abundant. UGRA refers to hen fed with Enterococcus faecalis UGRA10 at 108 Colony Forming Units per grams of fodder; while 40 and 80 refers to the number of days after the experiment started. [file Data_Sheet_1.docx]

| 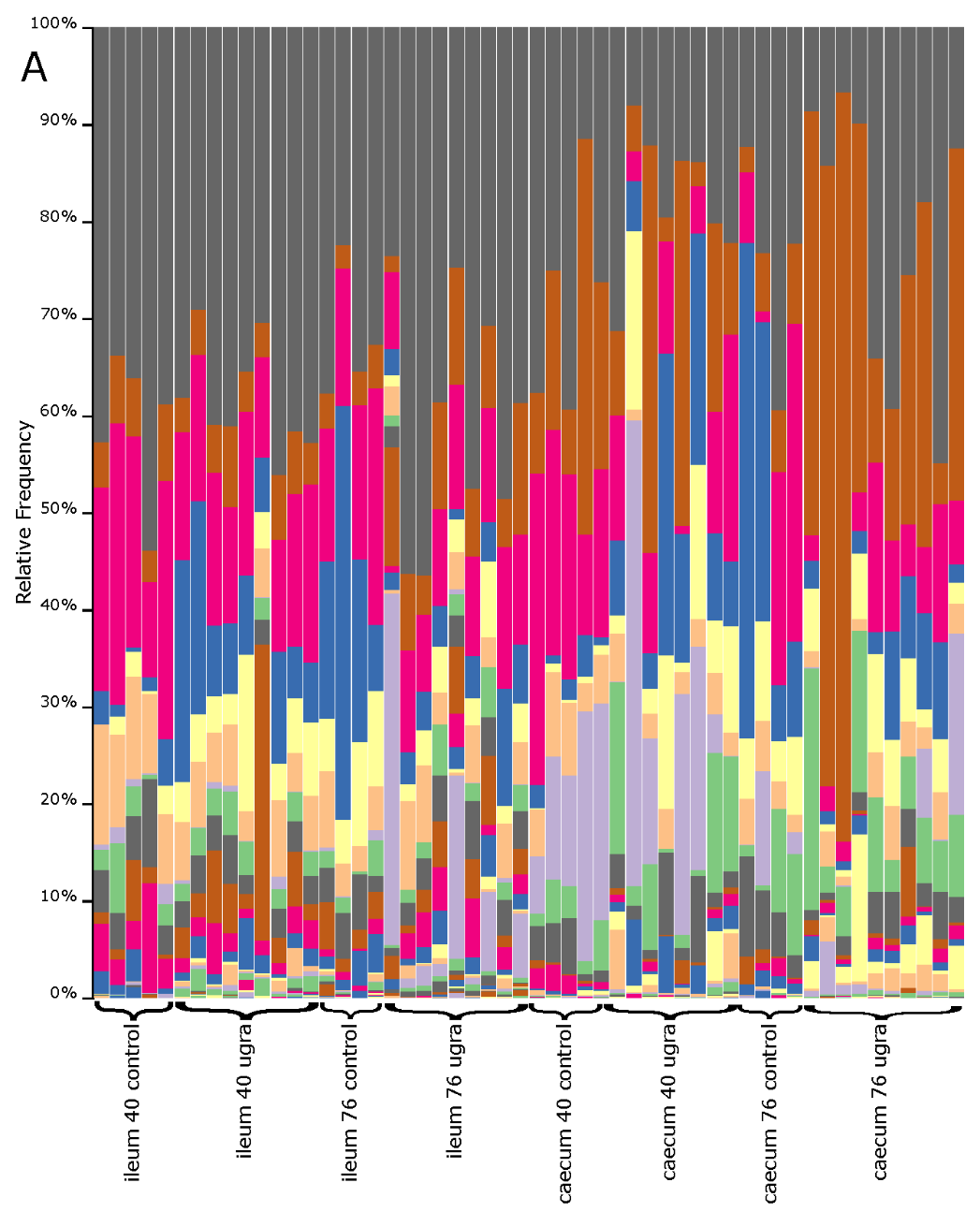 | 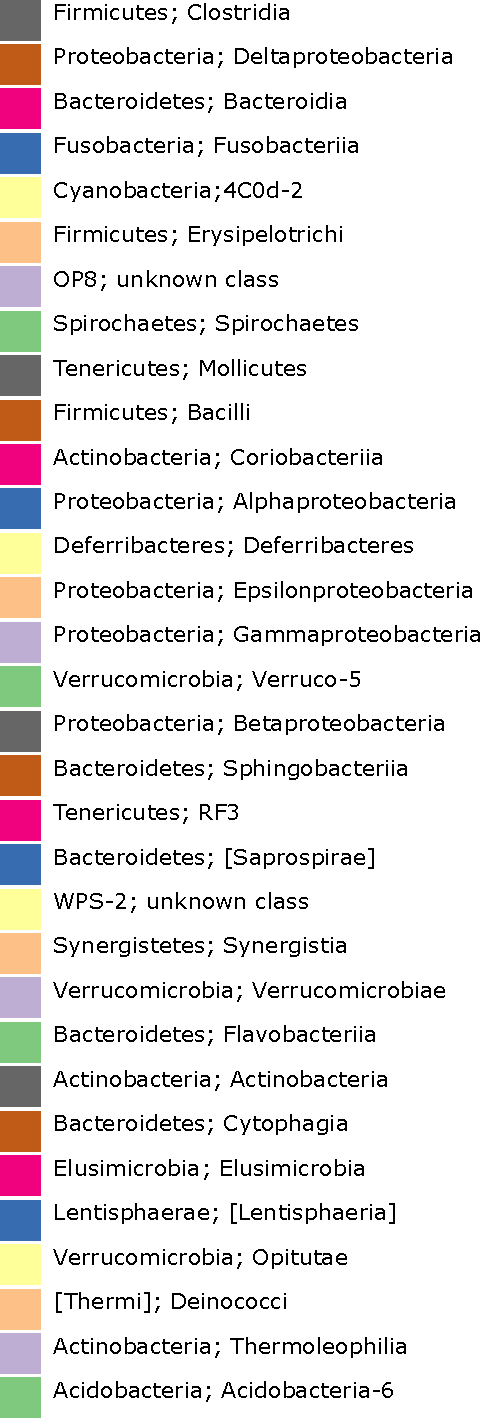 |
| --- | --- |

Figure S1. Bar plots of bacterial relative abundance at the class level in laying hens at different gut region, sampling times and treatments. Classes in the legend are sorted by sequence relative abundance, from most abundant to lowest abundant. UGRA refers to hen fed with *Enterococcus faecalis* UGRA10 at 10^8^ Colony Forming Units per grams of fodder; while 40 and 80 refers to the number of days after the experiment started.
