# Supplementary material for: Egg Production in Poultry Farming Is Improved by Probiotic Bacteria
Source: Front Microbiol. 2019 May 24;10:1042. doi: 10.3389/fmicb.2019.01042 (PMC6543855; doi:10.3389/fmicb.2019.01042)
Supplement: FIGURE S2 — Bar plots of relative bacterial abundance at the genus level of ileum and caecum in laying hens at different gut region (ileum and caecum), sampling times (30 and 80 days) and treatments (control and UGRA) (A) or grouped by gut region, sampling time and treatment (B). Classes in the legend are sorted by sequence relative abundance. UGRA refers to hen fed with Enterococcus faecalis UGRA10 at 108 Colony Forming Units per grams of fodder; while 40 and 80 refers to the number of days after the experiment started. [file Data_Sheet_2.docx]

| 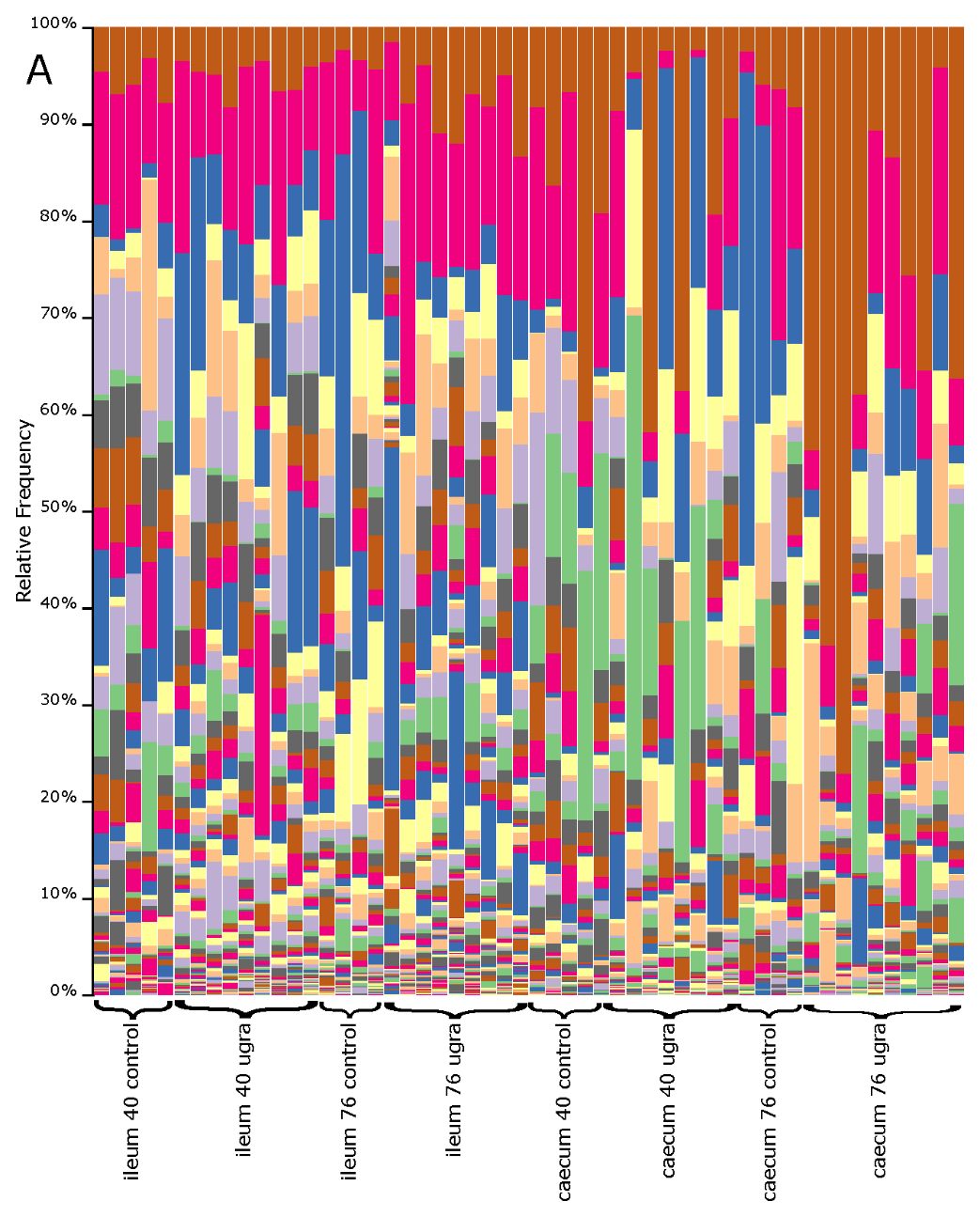 | 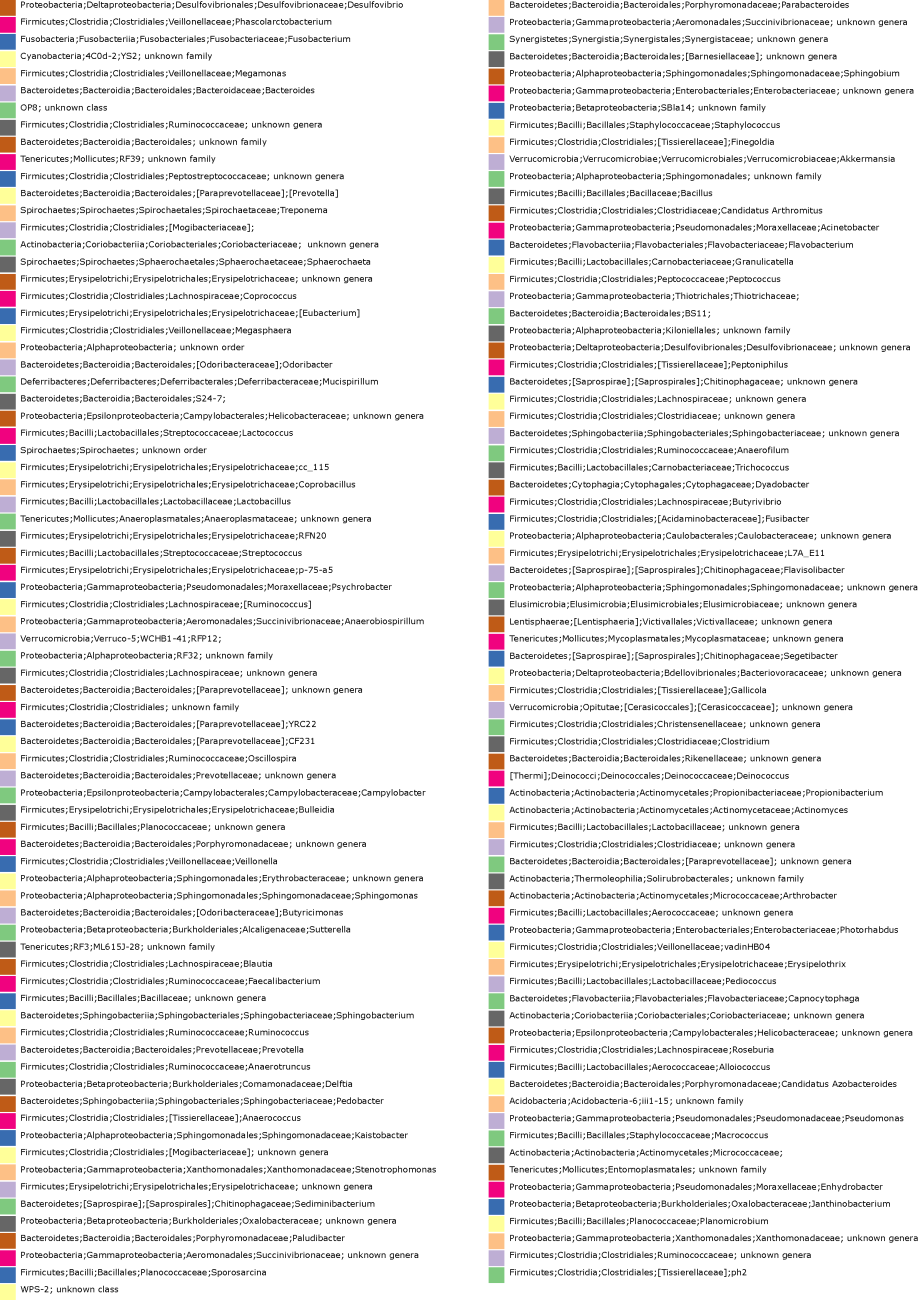 | 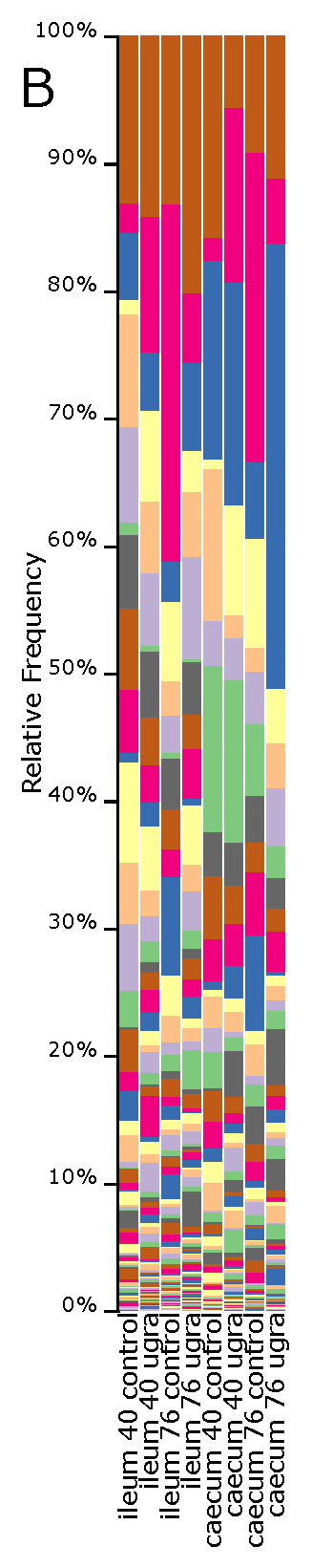 |
| --- | --- | --- |

FIGURE S2. Bar plots of relative bacterial abundance at the genus level of ileum and caecum in laying hens at different gut region (ileum and caecum), sampling times (30 and 80 days) and treatments (control and UGRA) (A) or grouped by gut region, sampling time and treatment (B). Classes in the legend are sorted by sequence relative abundance. UGRA refers to hen fed with *Enterococcus faecalis* UGRA10 at 10^8^ Colony Forming Units per grams of fodder; while 40 and 80 refers to the number of days after the experiment started.
