# Supplementary material for: Egg Production in Poultry Farming Is Improved by Probiotic Bacteria
Source: Front Microbiol. 2019 May 24;10:1042. doi: 10.3389/fmicb.2019.01042 (PMC6543855; doi:10.3389/fmicb.2019.01042)
Supplement: TABLE S1 — Sample sizes of bacterial community of ileum and caecum of laying hens, successfully amplified in Illumina MiSeq platform, at different sampling times. In parentheses, sample size before amplification. [file Table_1.docx]

TABLE S1. Sample sizes of bacterial community of ileum and caecum of laying hens, successfully amplified in Illumina MiSeq platform, at different sampling times. In parentheses, sample size before amplification.

|  | Day 40 | |  | Day 76 | |  |
| --- | --- | --- | --- | --- | --- | --- |
|  | Control | Treatment |  | Control | Treatment | Total |
| Ileum | 5 (5) | 9 (10) |  | 4 (5) | 9 (10) | 27 |
| Caecum | 5 (5) | 8 (10) |  | 4 (5) | 10 (10) | 27 |
