# Supplementary material for: Egg Production in Poultry Farming Is Improved by Probiotic Bacteria
Source: Front Microbiol. 2019 May 24;10:1042. doi: 10.3389/fmicb.2019.01042 (PMC6543855; doi:10.3389/fmicb.2019.01042)
Supplement: TABLE S3 — Summary of average (±SE) of alpha diversity indexes based in bacterial community of ileum and caecum in laying hens sequenced by Illumina Miseq. Different indexes have been separated by gut region, sampling date and treatment. In treated group, laying hens were supplemented with Enterococcus faecalis UGRA10. Sample size is also shown. [file Table_3.docx]

TABLE S3. Summary of average (±SE) of alpha diversity indexes based in bacterial community of ileum and caecum in laying hens sequenced by Illumina Miseq. Different indexes have been separated by gut region, sampling date and treatment. In treated group, laying hens were supplemented with *Enterococcus faecalis* UGRA10. Sample size is also shown.

| Gut region | Sampling date | Experimental group | N | Species richness | Pielou evenness | Faith’s diversity index | Shannon’s diversity index |
| --- | --- | --- | --- | --- | --- | --- | --- |
| Ileum | 40 days | Treatment | 9 | 237.00 (8.13) | 0.76 (0.01) | 27.84 (0.80) | 5.98 (0.12) |
|  |  | Control | 5 | 160.00 (8.24) | 0.79 (0.01) | 20.25 (1.07) | 5.79 (0.10) |
|  | 76 days | Treatment | 9 | 224.67 (12.50) | 0.74 (0.02) | 26.64 (1.13) | 5.76 (0.16) |
|  |  | Control | 4 | 207.50 (22.86) | 0.73 (0.03) | 24.84 (3.17) | 5.56 (0.30) |
| Caecum | 40 days | Treatment | 8 | 167.00 (18.26) | 0.67 (0.03) | 21.05 (2.00) | 4.95 (0.31) |
|  |  | Control | 10 | 159.40 (13.73) | 0.63 (0.04) | 20.62 (1.13) | 4.61 (0.32) |
|  | 76 days | Treatment | 5 | 156.60 (14.28) | 0.68 (0.03) | 20.19 (1.24) | 4.98 (0.30) |
|  |  | Control | 4 | 147.50 (11.47) | 0.69 (0.02) | 17.76 (1.33) | 4.96 (0.21) |
